# Six-Month Outcomes of a Web-Based Intervention for Users of Amphetamine-Type Stimulants: Randomized Controlled Trial

**DOI:** 10.2196/jmir.3778

**Published:** 2015-04-29

**Authors:** Robert J Tait, Rebecca McKetin, Frances Kay-Lambkin, Bradley Carron-Arthur, Anthony Bennett, Kylie Bennett, Helen Christensen, Kathleen M Griffiths

**Affiliations:** ^1^National Drug Research InstituteFaculty of Health SciencesCurtin UniversityPerthAustralia; ^2^National Institute for Mental Health ResearchThe Australian National UniversityCanberraAustralia; ^3^Centre for Research on Ageing, Health and WellbeingThe Australian National UniversityCanberraAustralia; ^4^National Drug and Alcohol Research CentreUniversity of New South WalesSydneyAustralia; ^5^Centre for Translational Neuroscience and Mental HealthUniversity of NewcastleNewcastleAustralia; ^6^Black Dog InstituteUniversity of New South Wales and Prince of Wales HospitalSydneyAustralia

**Keywords:** amphetamine-related disorders, Internet, randomized controlled trial, intervention studies, cognitive therapy

## Abstract

**Background:**

The use of amphetamine-type stimulants (ATS) places a large burden on health services.

**Objective:**

The aim was to evaluate the effectiveness of a self-guided Web-based intervention (“breakingtheice”) for ATS users over 6 months via a free-to-access site.

**Methods:**

We conducted a randomized trial comparing a waitlist control with a fully automated intervention containing 3 modules derived from cognitive behavioral therapy and motivation enhancement. The main outcome was self-reported ATS use in the past 3 months assessed at 3- and 6-month follow-ups using the Alcohol, Smoking, and Substance Involvement Screening Test (ASSIST). Secondary outcomes were help-seeking intentions (general help-seeking questionnaire), actual help seeking (actual help-seeking questionnaire), psychological distress (Kessler 10), polydrug use (ASSIST), quality of life (European Health Interview Survey), days out of role, and readiness to change. Follow-up data were evaluated using an intention-to-treat (ITT) analysis with a group by time interaction.

**Results:**

We randomized 160 people (intervention: n=81; control: n=79). At 6 months, 38 of 81 (47%) intervention and 41 of 79 (52%) control participants provided data. ATS scores significantly declined for both groups, but the interaction effect was not significant. There were significant ITT time by group interactions for actual help seeking (rate ratio [RR] 2.16; *d*=0.45) and help-seeking intentions (RR 1.17; *d*=0.32), with help seeking increasing for the intervention group and declining for the control group. There were also significant interactions for days completely (RR 0.50) and partially (RR 0.74) out of role favoring the intervention group. However, 37% (30/81) of the intervention group did not complete even 1 module.

**Conclusions:**

This self-guided Web-based intervention encouraged help seeking associated with ATS use and reduced days out of role, but it did not reduce ATS use. Thus, this program provides a means of engaging with some sections of a difficult-to-reach group to encourage treatment, but a substantial minority remained disengaged.

**Trial Registration:**

Australian and New Zealand Clinical Trials Registry: ACTRN12611000947909; https://www.anzctr.org.au/Trial/Registration/TrialReview.aspx?id=343307 (Archived by WebCite at http://www.webcitation.org/6Y0PGGp8q).

## Introduction

Methamphetamine and other amphetamine-type stimulants (ATS) increase the concentrations of monoamine neurotransmitters in the synaptic cleft with associated feelings of increased energy, confidence, and euphoria in the user [1]. It is estimated that between 14 and 52 million people used ATS in 2010. This means that the prevalence of ATS use is second only to cannabis of the illicit drugs [2]. ATS can be highly addictive, particularly the more potent formulations (eg, crystalline) and when used via more rapidly absorbed routes (eg, smoking, injecting) [3]. The prevalence of dependence on ATS and the harms resulting from ATS place a considerable burden on health resources, especially in Asia and Oceania, but also in Europe and North America [2,4].

In Australia, it is estimated that 97,000 people are dependent on ATS but few specialist treatment services are available [5]: data suggest that just 16% of nondependent and approximately 30% of dependent methamphetamine users received any treatment for their drug use in the previous year [6,7]. With no pharmacotherapy currently approved for ATS disorders, treatment relies on face-to-face interventions, typically in the form of cognitive behavioral therapy (CBT) or contingency management, which can be extremely resource intensive, preventing their widespread implementation [8,9].

Even where services exist, they are not always accessed by clients. Overall, approximately 24% of those with a substance use disorder used health services for a mental health problem in the previous year, but service use by young people, particularly young males, was much lower (13.2%) [10,11]. A number of potential physical and psychological barriers have been identified that could inhibit utilization of services, including cost, stigma, lack of awareness, and poor access [12,13]. Web-based interventions have the potential to extend the reach of conventional interventions and could overcome many of these impediments [14].

There has been considerable interest in the development and evaluation of Web-based interventions for tobacco use or alcohol consumption, with a recent systematic review summarizing data from studies involving nearly 40,000 smokers [15] and a review of online interventions for alcohol finding 16 studies comprising more than 5600 participants [16]. However, the development of Internet interventions for illicit drug use is at a more formative stage. A meta-analysis of outcomes for computer- and Web-based interventions for cannabis use reported on 10 studies with a total of 4125 participants. Overall the effect size in reducing consumption was small (*g*=0.16) [17]. Limited data are available for Web- and computer-based interventions for users of other illicit drugs, either targeting users of specific drugs (eg, cocaine) [18] or multiple drugs [19-22]. The authors are not aware of any other interventions specifically targeting users of ATS, although we have previously reported outcomes to 3 months [23].

The current study used a randomized design to evaluate the effectiveness of a Web-delivered fully automated intervention for users of ATS against a waitlist comparison group. It was hypothesized that the intervention group would have a greater reduction in their use of ATS 6 months after starting the intervention than the control group. We also examined whether the intervention resulted in improvements on a range of secondary outcomes (detailed subsequently).

## Methods

### Design

To evaluate the intervention, we used a randomized controlled trial with the intervention group receiving a fully automated, 3-module, Web-delivered intervention. Those in the waitlist group underwent the same assessments as the intervention group, but could not access the intervention for 6 months. Contact details for emergency services were given to participants for crisis support. We have previously described the methodology in detail; the key features are described in the following subsections [24].

### Sample

**Figure 1:**
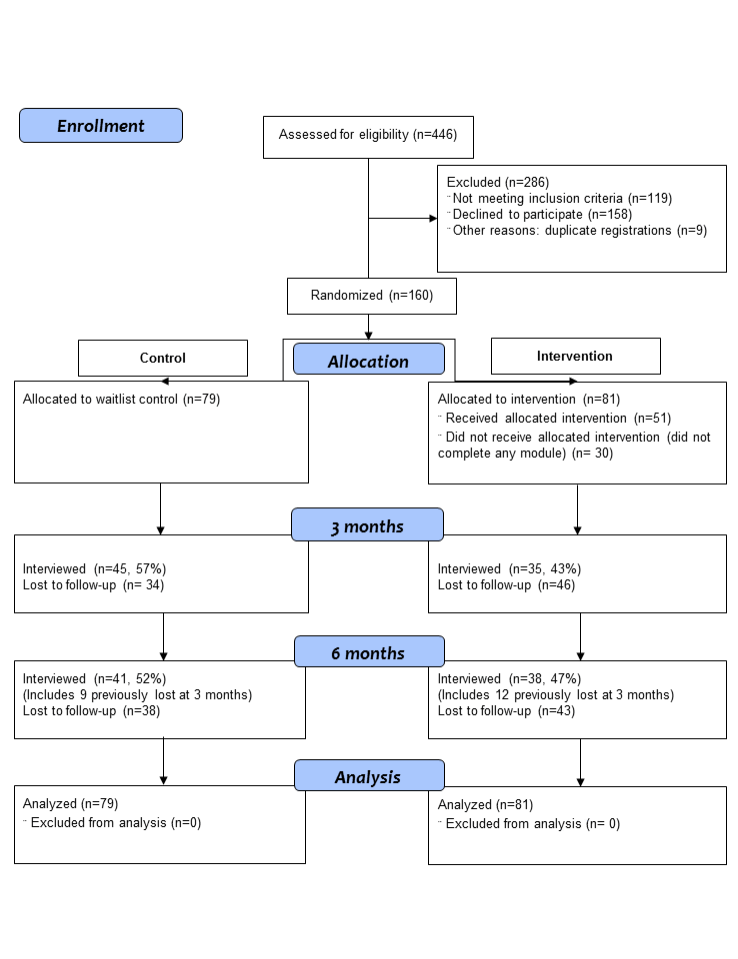
CONSORT flow diagram for breakingtheice study.

We advertised for participants on social network sites and posters in local clinics; all enrollment processes occurred through the study website. To be eligible, participants had to be resident in Australia, aged 18 years or older, and reported use of ATS (eg, meth/amphetamine, ecstasy, nonmedical use of prescription stimulants) in the previous 3 months. Because of the nature of the intervention, participants were required to have access to the Internet and to provide a valid email address. We excluded potential participants if they reported that they were currently receiving any treatment for stimulant abuse/dependence or methadone, naltrexone, or buprenorphine for a substance use disorder. Those who reported that a doctor had ever diagnosed them as having schizophrenia, schizoaffective, or bipolar disorder were also excluded. Finally, we inspected registration details and 9 cases were excluded as duplicate registrations (eg, identical IP addresses/payment addresses.). Figure 1 provides details of the number recruited and the flow of participants through the study to 6 months. Recruitment ran from January to July 2013.

### Procedure

All participants were screened and enrolled via the free study website. Those who fulfilled the eligibility criteria were invited to provide consent by “clicking” an onscreen box for each element of the consent form. After consent, the website generated a personalized link that was sent to the participant’s email address. This enabled the participants to create their own study username and password. Next, participants were directed to an online baseline survey. Finally, they were randomized using a fully automated system (1-to-1 allocation ratio and with permuted blocks of 4). Participants who were not eligible for the study were provided with information about other potentially relevant websites and community resources.

The intervention group was provided with immediate access to the first module. We recommended that 1 module should be completed each week, but participants were able at advance at their own pace. However, to progress through the program, each page of a module had to be “opened” in sequence to finish that module and progress to the next. Participants could return to any page or module that had already been accessed. Participants received a reminder email 3 days after the expected start date if the first module had not commenced, with a further email sent at day 7 when the next module was due. This pattern of emails was repeated for the other modules. Participants were sent further emails at 3 and 6 months inviting them to complete the follow-up surveys. There was also opportunity at 6 months to provide feedback on the intervention. Participants received AU $20 for each baseline and follow-up assessment, with payment by either posted or online vouchers. The Australian National University Human Research Ethics committee approved the study and it was registered with the Australian and New Zealand Clinical Trials Registry (#12611000947909).

### Measures

Outcome data were collected at 3 and 6 months and were all self-reported. The study’s primary outcome measure was ATS use assessed with the Alcohol, Smoking, and Substance Involvement Screening Test (ASSIST) [25]. We also collected secondary outcomes on (1) help-seeking intentions (General Help-Seeking Questionnaire) [26], (2) actual help seeking (Actual Help-Seeking Questionnaire) [27,28], (3) psychological distress (Kessler Psychological Distress Scale [K-10]) [29], (4) polydrug use measured by the ASSIST [25], (5) quality of life (European Health Interview Survey Quality of Life scale [EUROHIS]) [30], (6) days partial or wholly out of role [31], and (7) readiness to change (Readiness to Change Questionnaire [RTCQ]) [32]. Demographic details (eg, age, sex), history of drug use (eg, age of first use of ATS), and severity of dependence as measured by the Severity of Dependence Scale (SDS) [33] were collected as part of the baseline survey. The feedback survey included free-text fields plus the 16-item Internet Intervention Adherence Questionnaire [34] and the 16-item Satisfaction with Service measure adapted from the ANU Wellbeing study to reference ATS use rather than depression [35].

### Scoring and Coding

The ASSIST appraises lifetime and past 3-month use of 9 classes of drugs (ie, tobacco, alcohol, cannabis, cocaine, ATS, inhalants, sedatives, hallucinogens, opioids, and other). The standard ASSIST scoring algorithm was used to calculate a score for ATS use in the past 3 months, providing scores in the range 0-39 [25] (see Multimedia Appendix 1 for further information). We assessed help-seeking intentions with the question: “How likely is it that you would seek help from each of the following people for any amphetamine or other drug use problems during the next 4 weeks?” For each of 9 potential sources of help, there was a 7-point scale (“extremely unlikely” to “extremely likely”) with a score range between 9 and 63. The list did not include breakingtheice. The actual help-seeking measure asked “Which of the following people have you gone to for advice or help in the past 2 weeks for any amphetamine or other drug use problems?” It then listed the same 9 sources of help (range 0-9). We used the total score on the K-10 to evaluate psychological distress. The K-10 uses a 5-point scale (“none of the time” to “all of the time”) with a score range between 10 and 50 [29]. To quantify the extent of polydrug use, we summed the ASSIST classes of drugs, excluding ATS use [25]. The total score on the EUROHIS (range 8-40) was used to assess quality of life: higher scores indicate better outcomes [30]. We assessed days completely and partially out of role (both range 0-30) in the previous month using the wording from Kessler’s days out of role measure, but referencing “ATS drug use” rather than “depression” [31]. We modified the RTCQ to reference ATS as opposed to alcohol consumption. The measure has 4 items addressing the precontemplation, contemplation, and action stages. Participants were allocated to their highest scoring stage or, in the event of tied scores, to the higher stage [32].

### Sample Size

We determined the sample size required to evaluate the primary outcome (ATS score) at a power of 0.8 to detect a medium effect size (eg, *d*=0.5) [36]. This required 60 people per group, but allowing for 20% attrition, we recruited a total of 80 people per group. The effect size was based on the development study for the ASSIST in Australia [25] and a brief motivational intervention for non-treatment-seeking users of ecstasy [37].

### Modules

The content of the modules has been previously described [24]. We based the intervention on principles from motivational interviewing (MI) and CBT and adapted from a face-to-face intervention evaluated in amphetamine users [38]. We also adapted the “decisional balance” approach [39] and asked participants to list both the pros and cons of ATS consumption and the potentially good and bad outcomes anticipated from changing their use of ATS. To help participants change their drug use, we assisted them in the development of skills and strategies to aid in behavioral change (eg, identifying people who could assist them, approaches to help in controlling urges and overcoming cravings, refusal skills, and an action plan to deal with high-risk situations). Example images from the program are available in Multimedia Appendix 2.

### Analysis

Initial analyses were conducted in SPSS v21 (IBM Corp, Armonk, NY, USA). Descriptive analyses reported means with standard deviations for continuous measures or percentages for categorical outcomes together with their associated statistics (*t* test or chi-square test). Effect sizes were calculated as (1) difference in posttest minus pretest means for the 2 conditions divided by their common pretest standard deviation, multiplied by a bias correction factor (1–3/4[n_treatment_+n_control_-2]-1) [40] and (2) as Cohen’s *d* (posttest intervention mean minus posttest control mean divided by common standard deviation). The characteristics of participants lost to follow-up at 6 months was assessed with logistic regression using baseline predictors of condition, highest education level, age, age of first ATS use, gender, SDS, K-10, ASSIST ATS, polydrug use, RTCQ category, and actual and intended help-seeking scores.

The primary analysis used an intention-to-treat (ITT) approach with the effect of the intervention on each outcome being assessed using a time by group interaction. To analyze the correlated data arising from the repeated measures we used a multilevel mixed-effects regression model with a random intercept term to control for clustering of variance on individuals over repeated measures [41]. This analysis was conducted with Stata SE version 11.2 (StataCorp LP, College Station, TX, USA) using the xtmixed, xtmepoisson, and xtmelogit command suites for linear, Poisson, and logit models, respectively. Measures of days out of role, intended help seeking, and actual help seeking were analyzed using a Poisson distribution. Readiness to change was recoded as a binary variable reflecting action stage versus contemplation or precontemplation stages and analyzed using a logit model. All other outcomes were continuous and analyzed using a linear model. For all measures, we used an unstructured correlation matrix. At baseline, the groups differed significantly on actual help seeking (see Results). To adjust for this difference, baseline actual help seeking was included as a covariate in all models (except for where actual help seeking was the outcome). All models were adjusted for baseline SDS score due to its importance in predicting attrition (see Results).

For the primary outcome (ATS score), we imputed missing data using an iterative Markov chain Monte Carlo (MCMC) method in SPSS to generate 25 sets of data. Maximum and minimum values were logically constrained (eg, to the possible range of scores on the ASSIST), with baseline outcomes and demographic variables used as predictors. The imputed model was conducted in SPSS using the equivalent multilevel mixed-effects linear model to the unimputed model. We also conducted a per-protocol analysis where the “group” variable was replaced with a variable representing exposure to the intervention (“completed any modules,” “completed no modules,” or “control group”).

## Results

The majority of participants were male (121/160, 75.6%), the mean age was 22.4 (SD 6.3) years, and 18 of 160 (11.3%) reported using ATS daily or almost daily. In addition, previous treatment for ATS use was reported by 9.4% (15/160) of participants (control: n=7; intervention: n=8) and 23 of 160 (14.4%) reported ever injecting drugs. Table 1 displays the descriptive data at 6 months plus the effect sizes. Baseline characteristics were similar on all measures except for actual help seeking, in which the intervention group had significantly lower levels than the control group (mean 0.3 vs 0.8). (Multimedia Appendix 3 provides mean, SD, and n for each of the outcome variables).

**Table 1:** Descriptive characteristics by study group at baseline and 6 months plus effect sizes (change from baseline to 6 months on mean scores and between groups at 6 months).

Variable^a^		Baseline		6 months^b^		Effect size, *d*	
		Control n=79	Intervention n=81	Control n=41	Intervention n=38	From 0-6 months	Between groups at 6 months
Sex (male), n (%)		57 (72)^c^	64 (79)				
Age (years), mean (SD)		22.5 (7.1)	22.2 (5.5)				
Age first ATS use, mean (SD)		18.6 (4.2)	17.7 (2.6)				
SDS, mean (SD)		3.8 (3.3)	3.7 (3.5)				
**ATS frequency in past 3 months, n (%)**							
	Never	—	—	8 (20)	5 (13)		
	1-2 times	27 (34)	20 (25)	12 (29)	14 (37)		
	Monthly	18 (23)	33 (41)	9 (22)	9 (24)		
	Weekly	23 (29)	21 (26)	10 (24)	6 (16)		
	Daily/almost daily	11 (14)	7 (9)	2 (5)	4 (11)		
ATS score, mean (SD)		16.8 (11.1)	17.0 (10.1)	12.8 (11.1)	13.8 (9.6)	0.07^d^	0.10^d^
Intended help seeking, mean (SD)		20.4 (10.9)	19.7 (11.2)	19.4 (9.2)	22.6 (12.3)	0.32^e^	0.31^e^
Actual help seeking, mean (SD)		0.8 (1.3)^f^	0.3 (0.7)^f^	0.6 (0.9)	0.6 (0.9)	0.45^e^	—
Any actual help seeking (yes), n (%)		34 (43)	20 (25)	15 (37)	16 (42)		
K-10 score, mean (SD)		22.3 (8.3)	22.2 (8.4)	22.0 (8.7)	22.9 (10.0)	0.12^d^	0.10^d^
Polydrug use, mean (SD)		4.6 (1.6)	4.8 (1.8)	4.4 (1.9)	4.5 (2.1)	–0.06^e^	0.05^d^
Quality of life, mean (SD)		28.2 (5.8)	27.2 (6.3)	28.6 (6.8)	27.3 (6.8)	0.05^d^	0.19^d^
Days out of role, mean (SD)		2.9 (5.9)	3.5 (5.6)	2.9 (5.8)	2.8 (6.2)	–0.12^e^	0.02^e^
Days partially out of role, mean (SD)		3.2 (4.8)	3.9 (5.3)	2.8 (4.5)	3.3 (5.7)	–0.04^e^	0.05^d^
**RTCQ, n (%)**							
	Precontemplation	32 (41)	27 (33)	17 (42)	13 (34)		
	Contemplation	24 (30)	35 (43)	9 (22)	7 (18)		
	Action	23 (29)	19 (24)	15 (37)	18 (47)		

^a^ ATS: amphetamine-type stimulants; K-10: Kessler 10; quality of life: EUROHIS score; RTCQ: Readiness to Change Questionnaire; SDS: Severity of dependence.

^b^ Missing data at 6 months all outcome variables missing data n=81

^c^ One person reported sex as “other”.

^d^ Favors control group.

^e^ Favors intervention group.

^f^ Levene’s correction for inequality of variances (*t*_113_=2.83, *P*=.01).

### Attrition and Engagement

At 6 months, 41 of 79 (52%) participants from the control and 38 of 81 (47%) from the intervention completed follow-up surveys (Figure 1). Logistic regression showed that retention was not significantly related to group allocation (OR 1.17, 95% CI 0.56-2.47). However, females had higher odds of retention (OR 3.11, 95% CI 1.28-7.55) as did older participants (OR 1.10, 95% CI 1.00-1.20) and those with greater psychological distress (K-10 scores; OR 1.07, 95% CI 1.01-1.14). In addition, higher baseline SDS scores reduced the odds of remaining in the study (OR 0.73, 95% CI 0.59-0.91). In terms of “exposure to the intervention” among the intervention group, 30 of 81 (37%) people did not start or complete the first module, 6 of 81 (7%) completed 1 module only, 6 of 81 (7%) completed 2 modules only, and 39 of 81 (48%) completed all 3 modules. Those who completed any modules (28/51) were not more likely to complete the 6-month follow-up than those who completed zero modules (10/30; χ^2^_1_=3.5, *P*=.06).

### Outcomes

There was a significant main effect on ATS scores, with both groups reducing use by 6 months (b=*–*2.59, SE 0.98; *P*=.008). However, the interaction term was not significant, showing that the intervention group did not improve more than the control group (Table 2). The ITT analysis was based on those with baseline data plus at least 1 follow-up. There was a significant group by time interaction for actual help seeking (b=0.77*, P*=.02, rate ratio [RR] 2.16, 95% CI 1.14-4.10) and for intended help seeking (RR 1.17, 95% CI 1.05-1.31). There were also significant group by time interactions for number of days out of role (RR 0.50, 95% CI 0.37-0.68) and days partially out of role (RR 0.74, 95% CI 0.56-0.98). In both instances, the intervention group had a greater reduction in days of “impairment” than the control group. Finally, a greater proportion of those receiving breakingtheice transitioned to the action stage than controls (OR 4.13, 95% CI 1.03-16.58). In the analyses involving imputation, the overall group by time interaction for ATS scores was not significant (*F*_1,318_=0.165, *P*=.69) controlling for baseline SDS and baseline actual help seeking.

**Table 2:** Statistics for group by time interaction^a^ for intention-to-treat (ITT) analyses with unstandardized coefficient (b), standard error (SE), and *P* values.

Variable^b^	Group × 3 and 6 months		Group × 3 months		Group × 6 months	
	b (SE)	*P*	b (SE)	*P*	b (SE)	*P*
ATS score	0.27 (1.41)	.85	0.10 (1.67)	.95	0.87 (1.91)	.65
Intended help seeking	0.16 (0.06)	.005	0.01 (0.07)	.87	0.28 (0.07)	<.001
Actual help seeking	0.77 (0.33)	.02	0.61 (0.39)	.12	0.90 (0.40)	.02
K-10 score	–0.16 (1.20)	.90	–1.79 (1.26)	.16	0.81 (1.71)	.64
Polydrug use	–0.37 (0.30)	.23	–0.64 (0.36)	.08	–0.16 (0.37)	.68
Quality of life	0.52 (0.85)	.55	0.75 (0.95)	.43	0.46 (1.15)	.69
Days out of role	–0.70 (0.16)	<.001	–1.05 (0.24) *p*	<.001	–0.72 (0.18)	<.001
Days partially out of role	–0.30 (0.14)	.04	–0.53 (0.18)	.003	–0.19 (0.17)	.27
RTCQ	1.42 (0.71)	.045	1.04 (0.76)	.17	1.61 (0.93)	.08

^a^ Group by time interactions adjusted for actual help seeking at baseline and SDS score at baseline: reference group=control.

^b^ ATS: amphetamine-type stimulants; K-10: Kessler 10; quality of life=EUROHIS score; RTCQ: action stage on the Readiness to Change Questionnaire.

### Effect of Exposure

The “per-protocol” analyses found significant condition (completed any modules, completed no modules, control group) by time interactions for a number of variables for those who completed any modules compared with controls. For actual help seeking, the rate ratio was 3.13 (95% CI 1.43-6.84) and for intended help seeking it was 1.31 (95% CI 1.16-1.48). Those who completed any modules had significant reductions in both days out of role (RR 0.46, 95% CI 0.33-0.63) and partial days out of role (RR 0.57, 95% CI 0.41-0.77) compared with controls. Those completing any modules were also more likely to transition to the action stage than controls (OR 7.22, 95% CI 1.45-36.01). In one instance, there was a significant effect for those who did not complete any modules compared with controls—those taking no modules had an increase in days partially out of role compared to controls (RR 1.56, 95% CI 1.00-2.42). It should be noted that a per-protocol analysis no longer represents randomized data.

### Feedback on the Intervention

Of the 81 people randomized to the intervention, 35 (43%) provided feedback at 6 months. Free-text responses in particular identified the use of fictional case stories as an engaging approach. The main criticisms included the assumption that people wanted to change their behavior and the lack of information on benefits of drug use (eg, use of ATS to control symptoms of attention deficit hyperactivity disorder). The most frequently cited negative reactions to the intervention were concerns about privacy (16/35, 46%) and boredom (7/35, 20%). Most participants (22/35, 63%) reported that using the intervention had reduced their adverse drug effects, 86% (30/35) would recommend the site, 86% (30/35) endorsed Internet delivery, 91% (32/35) rated the site as easy to use, and 91% (32/35) were satisfied with the program.

## Discussion

### Principal Findings

The results of this study suggest that this fully automated Web-based intervention may be useful both to increase help seeking among people who use ATS and to augment their intention to seek help in the future. There was also evidence for a reduction in the number of days completely and partially out of role. However, the intervention did not reduce ATS use relative to a waitlist control group. Furthermore, relative to the control group, there was no evidence that the intervention reduced the use of other drugs, improved quality of life, or reduced psychological distress.

We believe that breakingtheice is the first Web-based intervention specifically targeting ATS users. However, its lack of impact on ATS use is consistent with 2 previous Internet interventions that included stimulant users. Snow Control targeted cocaine use [18] and found a decline in milligrams of cocaine used per week at 6 months, but no significant effect of the intervention over an attention control group. However, the extremely high rate of attrition at 6 months (94%) makes extrapolation from their findings difficult. A generic online illicit drug (plus alcohol) screening and feedback program developed in Sweden also reported high attrition by 6 months with 69% lost to follow-up. Concordant with the current and Snow Control studies, although drug use declined for both the intervention and controls, there was no significant group by time interaction at 6 months for drug use in the Swedish trial [22].

In contrast, a recent Web-based intervention for cannabis use derived from CBT and MI principles was successful in reducing the frequency of cannabis use, but did not motivate participants to seek additional professional help for their cannabis use, with none of the experimental group seeking additional treatment by 3 months [42]. Although both the Rooke et al [42] and our study involved illicit drug users and adapted treatments from the same paradigms, the profiles of the participants differed with those in the cannabis study being older (mean age 31 years), having higher SDS (mean 14), and younger age of first use (mean 16 years). Further, the reduction in their cannabis use may mean that they did not feel that extra help was required.

The mechanism by which the intervention resulted in fewer days either partially or completely out of role is unclear given that there was not a concomitant decrease in ATS scores or improvements in quality of life or mental well-being. In our sample at baseline, the days out of role equated to 35-42 days per year, which is comparable with international data from high-income countries for drug abuse (mean 37.8 days/year) [43]. At 6 months, there was no change for the controls but the intervention group had fallen to 33.6 days per year, an improvement of more than 8 days per year. Subsequent research is required to identify a plausible mechanism for this change.

### Limitations

There are some limitations that should be considered in the interpretation of these results. Firstly, the loss of participants to follow-up threatens the validity and generalizability of conclusions based on these data. However, the rate of attrition (51%) is comparable with the average for fully automated interventions (47%) [44], even though substance users would be regarded typically as a group that is particularly difficult to retain in research projects and treatment. Yet, some study designs have improved follow-up, albeit with a different target group. Interventions that have recruited mother-daughter dyads to prevent cannabis use have achieved at least 90% retention at 12 months [17], but this method requires evaluation in other groups. In addition to loss to follow-up, the fact that a substantial minority in the intervention group did not complete even the first module is of concern, although a low level of engagement has also been identified as a difficulty in face-to-face interventions [45,46]. This low level of engagement may have contributed to the smaller that estimated effect size and the consequent null results. However, the potential for widespread dissemination means that even interventions with small effects can have a public health impact [47].

The representativeness of our sample of ATS users, who were required to have access to the Internet, compared with ATS users in general could be questioned. However, at least in Canada, it appears that users of cocaine or cannabis are as likely to have access to the Internet as current drinkers [48] and the Internet has been shown to be an effective means of reaching hidden populations [49]. Nevertheless, it seems probable that this approach will not reach the most severely disadvantaged ATS users.

Although the feedback on the site was generally positive, we note that these comments only represent a small proportion of the intervention group: we would anticipate that those lost to follow-up would be likely to have more negative opinions. We did not correct for multiple statistical testing, in particular for the secondary outcome measures (eg, all measures other than the ATS score) and, thus, interpret our findings cautiously. Nevertheless, we believe that the inclusion of a range of secondary outcomes is warranted given that this is the first intervention of this type with this population. With the caveat that per-protocol analyses are biased, the per-protocol analyses supports the interpretation that the changes in both help-seeking measures, in days out of role, and transition to the action stage on the RTCQ are not simply type 1 errors due to multiple statistical testing. In each case, it was those who had been exposed to the intervention that showed improvements, whereas those who did not complete at least 1 module showed no improvement compared with those in the control group.

We attempted to prevent duplicate registrations via inspection of IP addresses and payment addresses. This approach does not guarantee that duplicate registrations were eliminated with the potential that dynamic IP addresses or multiple sites could be used together with multiple email or physical addresses.

### Implications

The finding that the intervention did not reduce ATS use per se is unsurprising for several reasons. First, most of the intervention focused on enhancing people’s motivation to reduce their ATS use and seek help, with only the later modules focused on strategies for reducing ATS use (module 3). Most of the participants did not complete the last module. Second, the intervention was designed to attract a broad range of ATS users, but a desire to reduce or cease consumption of ATS was not a requirement of the study. With respect to alcohol use, screening and brief interventions in non-treatment-seeking groups have been found to be effective [50]. The majority of participants in this trial were using ATS at low levels (only 11% were using daily or almost daily). Indeed, the feedback on the intervention suggested that most people in the trial were not seeking to reduce their ATS use with less than one-third in the action phase (see Table 1) based on the RTCQ. This may explain why we failed to reduce ATS use. Implementation of the intervention with ATS users who had a greater need or desire for treatment would be required before dismissing its potential to impact on ATS use and related harms. Therefore, it may be necessary to further develop aspects of the module that specifically aim to reduce ATS use.

As noted previously, there was evidence of increased help seeking associated with the intervention, albeit that this was predominantly with informal sources of help. Nevertheless, outside the constraints of a research trial, any engagement with the program could be used as an opportunity to provide information and encouragement to seek further help, particularly for those with low levels of interaction with the online program. There is also the potential to evaluate the intervention as an adjunct to conventional face-to-face treatment. Previous research suggests that compared to a face-to-face CBT intervention alone, online interventions designed to reduce illicit drug use can be effective as an adjunct to weekly individual and group CBT [51]. Integration with face-to-face services could also provide the opportunity to allay privacy concerns expressed by some participants. Furthermore, a Web-based program might allow the extent of face-to-face treatment to be reduced and, hence, lower the burden on service providers and clients inherent in standard treatment.

### Conclusions

There is strong evidence for the effectiveness of technological-based programs such as Web- or computer-delivered interventions for problematic use of alcohol or tobacco use [52-55], but their impact on illicit drug use is less certain [14,17]. Nevertheless, this study demonstrates that is possible to engage some ATS users with a Web-based program and retain many of them in a trial to 6 months, but a substantial minority remained disengaged from the process and the effect size across range of measures was small.

## Multimedia Appendix 1

Scoring the ASSIST.

## Multimedia Appendix 2

BTI screenshots.

## Multimedia Appendix 3

CONSORT-EHEALTH checklist V1.6.2 [56].

## Abbreviations

ASSIST: Alcohol, Smoking, and Substance Involvement Screening Test
ATS: amphetamine-type stimulants
CBT: cognitive behavioral therapy
ITT: intention-to-treat
MCMC: Markov chain Monte Carlo
MI: motivational interviewing
RTCQ: Readiness to Change Questionnaire
SDS: Severity of Dependence Scale
