# Supplementary material for: Six-Month Outcomes of a Web-Based Intervention for Users of Amphetamine-Type Stimulants: Randomized Controlled Trial
Source: J Med Internet Res. 2015 Apr 29;17(4):e105. doi: 10.2196/jmir.3778 (PMC4430678; doi:10.2196/jmir.3778)
Supplement: Supplementary file 1 [file jmir_v17i4e105_app1.pdf]

Scoring the ASSIST

The exact question items and the scoring procedure for the ASSIST can be found in the report by Humeniuk et al [1]. In brief, the ATS score was the sum of six items. The first four items reference ATS use in the last 3 months while questions 5 and 6 reference lifetime use (the anchor items and values are shown in brackets).

1) Frequency of ATS use (never 0 – daily or almost daily 6)
2) Strong desire or urge to use (never 0 – daily or almost daily 6)
3) Use lead to health, social, legal or financial problems (never 0 – daily or almost daily 7)
4) Failed to do what was normally expected (never 0 – daily or almost daily 8)
5) Has a friend or relative ever expressed concern (No, never 0 – Yes, in the last 3 months 6)
6) Have you ever tried to cut down on using (No, never 0 – Yes, in the last 3 months 6)

1. Humeniuk R, Dennington V, Ali R, on behalf of the WHO ASSIST Phase III Study Group. The effectiveness of a brief intervention for illicit drugs linked to the alcohol, smoking and substance involvement screening test (ASSIST) in primary health care settings: a technical report of phase III findings of the WHO ASSIST randomized controlled trial. Geneva: World Health Organization; 2008. ISBN:9789241596510
