# Supplementary figures and images for: Six-Month Outcomes of a Web-Based Intervention for Users of Amphetamine-Type Stimulants: Randomized Controlled Trial
Source: J Med Internet Res. 2015 Apr 29;17(4):e105. doi: 10.2196/jmir.3778 (PMC4430678; doi:10.2196/jmir.3778)

## Slide 1
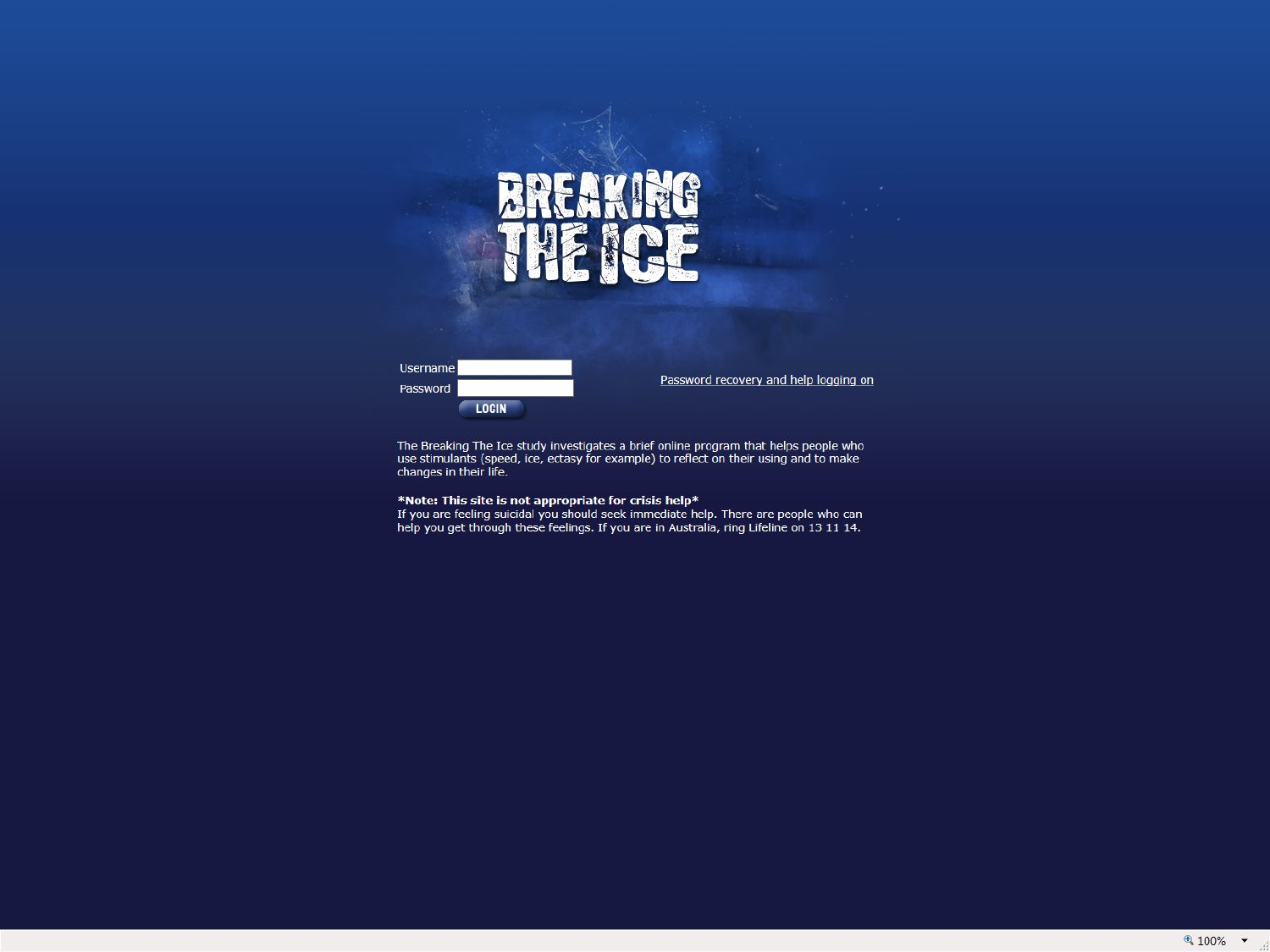

## Slide 2
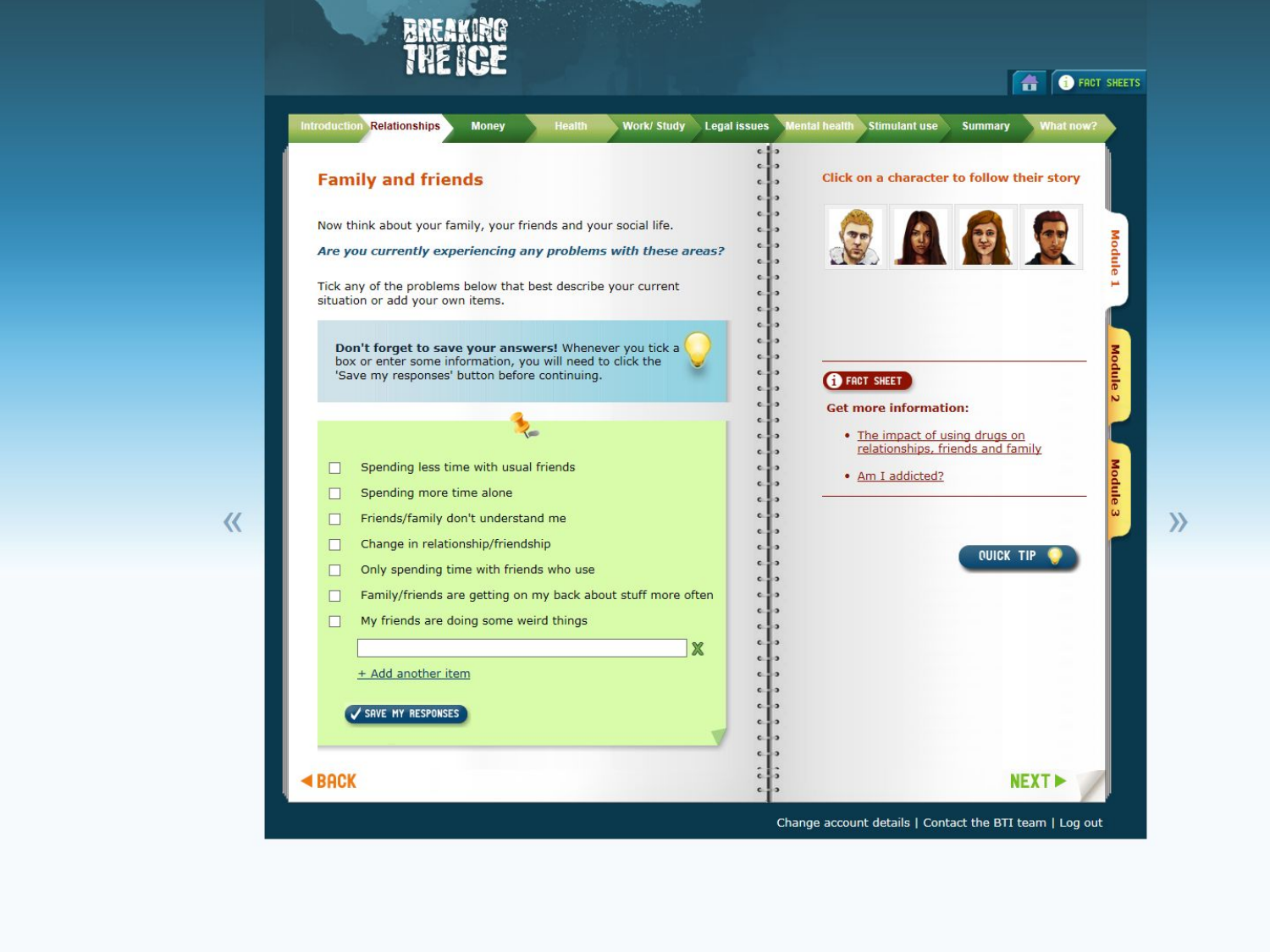

## Slide 3
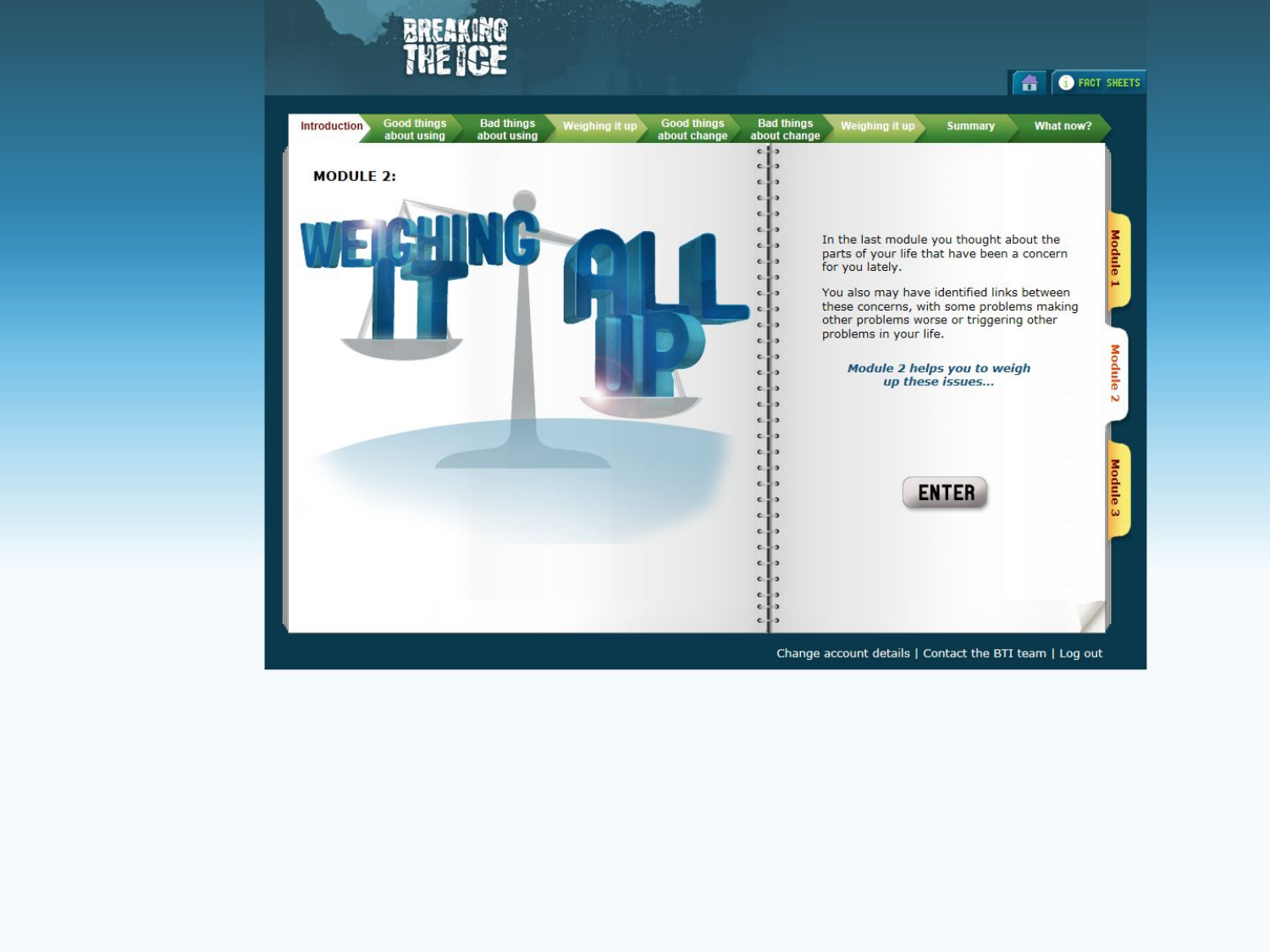

## Slide 4
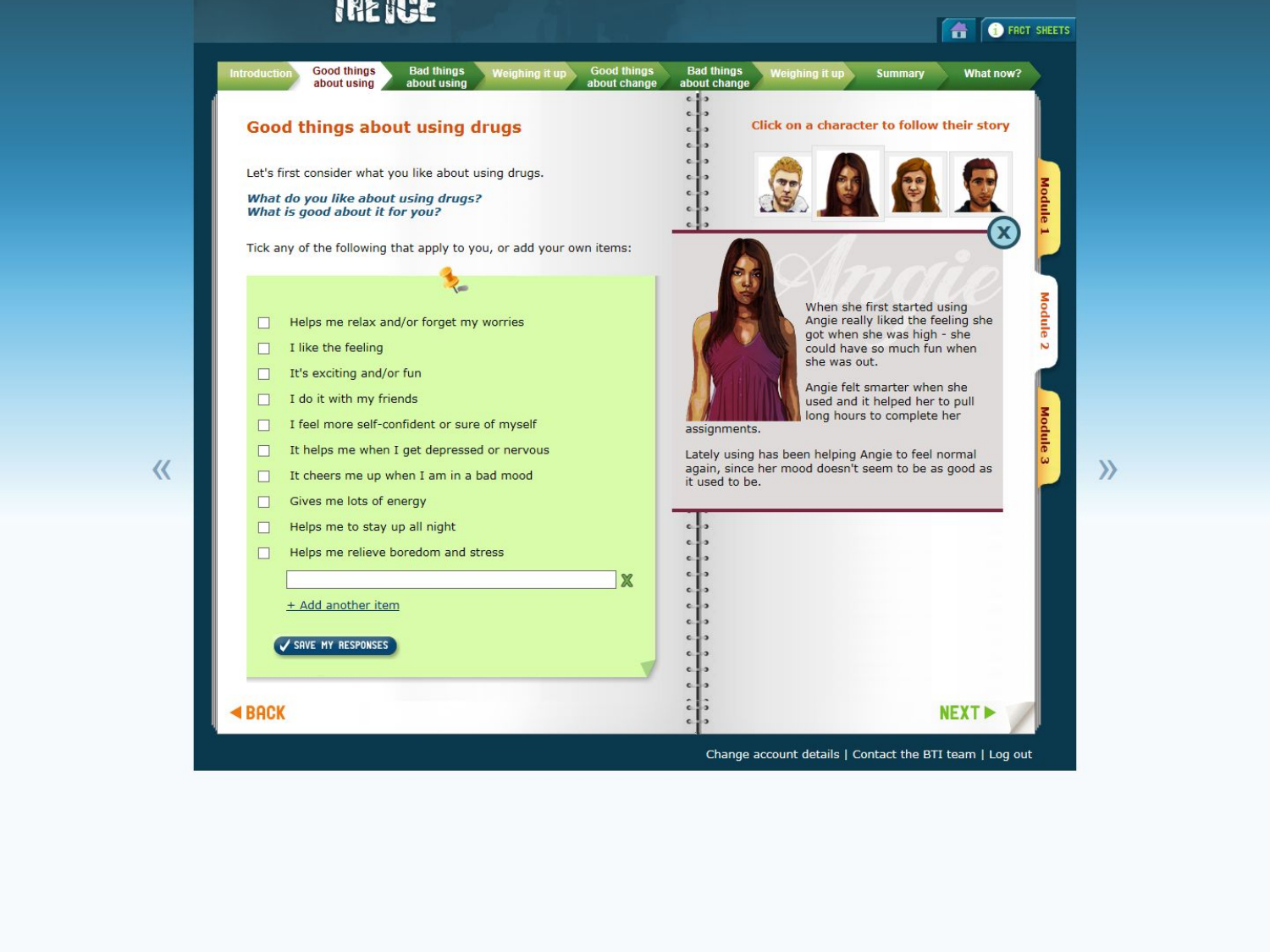

Supplement: Supplementary file 2 [file jmir_v17i4e105_app2.pptx]
