# Supplementary material for: Six-Month Outcomes of a Web-Based Intervention for Users of Amphetamine-Type Stimulants: Randomized Controlled Trial
Source: J Med Internet Res. 2015 Apr 29;17(4):e105. doi: 10.2196/jmir.3778 (PMC4430678; doi:10.2196/jmir.3778)
Supplement: Supplementary file 3 [file jmir_v17i4e105_app3.pdf]

CONSORT-EHEALTH Checklist V1.6.2 Report

(based on CONSORT-EHEALTH V1.6), available at [http://tinyurl.com/consort-ehealth-v1-6].

3778

Date completed
4/30/2014 0:27:10
by
Robert Tait

Breakingtheice: 3-month outcomes of a randomised controlled trial of a web-based intervention for users of amphetamine-type stimulants

TITLE

1a-i) Identify the mode of delivery in the title
randomised controlled trial of a web-based intervention
1a-ii) Non-web-based components or important co-interventions in title
not applicable
1a-iii) Primary condition or target group in the title
"users of amphetamine-type stimulants"

ABSTRACT

1b-i) Key features/functionalities/components of the intervention and comparator in the METHODS section of the ABSTRACT

"The intervention consisted of three fully automated, self-guided modules based on cognitive behavioral therapy and motivation enhancement."

"compared with a waitlist control group"
1b-ii) Level of human involvement in the METHODS section of the ABSTRACT
"fully automated"
1b-iii) Open vs. closed, web-based (self-assessment) vs. face-to-face assessments in the METHODS section of the ABSTRACT
"a free-to-access site" "primary outcome measure was self-reported "
1b-iv) RESULTS section in abstract must contain use data

1b-v) CONCLUSIONS/DISCUSSION in abstract for negative trials
"We randomized 160 people (intervention n = 81, control n = 79). At three months, 35 (43%) intervention and 45 (57%) control participants provided follow-up data. In the intervention group, 51 (63%) completed at least one module"

INTRODUCTION

2a-i) Problem and the type of system/solution
"Global assessments of illicit drugs place the prevalence of amphetamine type stimulant (ATS) use second only to cannabis ...., This use translates into ATS being listed as the primary drug of abuse for more than 20% of those in treatment in Asia ...most users do not reach diagnostic criteria. Therefore, interventions are needed across the spectrum from harm reduction for irregular 'recreational' use through to treatment of stimulant use disorders"
2a-ii) Scientific background, rationale: What is known about the (type of) system
"Currently, the treatment of ATS disorders relies on psychosocial interventions .... such as contingency management, cognitive behavior therapy (CBT) and motivational interviewing (MI)....traditional behavioral treatment options are not generally accessed by ATS users". " there is potential to develop web-delivered ... treatments for ATS users, an approach that has been effective with other conditions. The evidence base for the effectiveness of e-health interventions for illicit drug use is limited, and we are not aware of any e-health treatment interventions that currently exist specifically for ATS users"

METHODS

3a) CONSORT: Description of trial design (such as parallel, factorial) including allocation ratio
"The aim of the current study was to evaluate a fully automated, self-guided web-delivered intervention derived from established psychological approaches (i.e., CBT and MI) to reduce the use of ATS and associated problems at three months post-intervention."
3b) CONSORT: Important changes to methods after trial commencement (such as eligibility criteria), with reasons
No changes to report
3b-i) Bug fixes, Downtimes, Content Changes
No changes to report
4a) CONSORT: Eligibility criteria for participants

"We recruited participants by advertising on social networking sites and posters in local clinics"
"All stages of enrolment and screening were performed via the free study website"
4a-i) Computer / Internet literacy
"Given the nature of the intervention, participants required access to the internet and a valid e-mail address"
4a-ii) Open vs. closed, web-based vs. face-to-face assessments:
"All stages of enrolment and screening were performed via the free study website"
Participants were quasi anonymous so we attempted to exclude multiple registrations
"nine cases were excluded as duplicate registrations (e.g. duplicate IP addresses / e-mail addresses / payment addresses. Inspection of log files... indicated that these were likely to be repeated registrations)"
4a-iii) Information giving during recruitment
"Eligible participants provided active consent by 'clicking' on a box for each element of the consent form" Attached as appendix 1
4b) CONSORT: Settings and locations where the data were collected
" To be eligible, participants had to be resident in Australia,"
4b-i) Report if outcomes were (self-)assessed through online questionnaires
All the study measures were self-report.
4b-ii) Report how institutional affiliations are displayed
Not addressed specifically - the institution is clearly identified in the information / consent form (appendix 1) and the logo was on online advertising.
5) CONSORT: Describe the interventions for each group with sufficient details to allow replication, including how and when they were actually administered
5-i) Mention names, credential, affiliations of the developers, sponsors, and owners
"In developing the intervention we......"

5-ii) Describe the history/development process

This was detailed in the protocol paper which is referenced in the current manuscript.

5-iii) Revisions and updating

No updates or changes during the study.

5-iv) Quality assurance methods

Not done

5-v) Ensure replicability by publishing the source code, and/or providing screenshots/screen-capture video, and/or providing flowcharts of the algorithms used

"Sample images from the intervention are available elsewhere... and in appendix 2."

5-vi) Digital preservation

Only preserved via screenshots

5-vii) Access

"All stages of enrolment and screening were performed via the free study website. Eligible participants provided active consent by 'clicking' on a box for each element of the consent form (appendix 1). A personalized link to access the study was sent to verify their e-mail address and to allow them to create a username and password."
"Participants received AU$20 for baseline and follow-up assessments"

5-viii) Mode of delivery, features/functionalities/components of the intervention and comparator, and the theoretical framework

"In developing the intervention we drew on motivational enhancement and cognitive behavioral therapy methods that had been used in clinical practice with amphetamine users [18]. The approach was one of harm minimization, with participants able to decide on the most appropriate goals for themselves e.g. quitting completely, reducing their drug use, using in a less hazardous manner. Module one explores the typical problems which ATS users incur including: relationships with family and friends, health, finances, work/study, legal issues, mental health, and specific drug use problems. The last page provides a summary of the endorsed problems and guides the participant to generate a 'map' of the interconnections between these issues. The second module requires participants to think about the pros and cons of their stimulant use and the likely good and bad things related to changing their behavior, and draws on the Miller and Rollnick model [19]. To aid in their 'decision balance' for each element that they select, participants rate its importance. The last module focuses on behavioral change including techniques such as setting clearly specified goals, actions on specific dates, strategies to help with controlling and overcoming cravings, refusal skills, managing a 'slip', and an action plan to deal with high risk situations."

5-ix) Describe use parameters

"Those in the intervention group were given immediate access to the first module. Participants were advised to allow one week between modules, but could progress at their own pace although each page in a module had to be opened in sequence to complete the module and obtain access to the next one."

5-x) Clarify the level of human involvement

"The intervention consisted of three fully automated, self-guided modules .."
There were however "Reminder e-mails were sent three days after the scheduled start date if it had not been commenced, and at day seven when the next module was due. This was repeated for the third module. An e-mail invitation to complete the follow-up assessment was sent after three months."
These were computer generated (automated) and not tailored other than included a username.

5-xi) Report any prompts/reminders used

"Reminder e-mails were sent three days after the scheduled start date if it had not been commenced, and at day seven when the next module was due. This was repeated for the third module."

"An e-mail invitation to complete the follow-up assessment was sent after three months."

5-xii) Describe any co-interventions (incl. training/support)

Not applicable - fully automated

6a) CONSORT: Completely defined pre-specified primary and secondary outcome measures, including how and when they were assessed

"The primary outcome measure was ATS use evaluated with the Alcohol, Smoking, Substance Involvement Screening Test (ASSIST) [20]. The ASSIST assesses lifetime and last three-month use of nine drug categories (i.e. tobacco, alcohol, cannabis, cocaine, ATS, inhalants, sedatives, hallucinogens, opioids and other). Data includes frequency of use, cravings, problems (health, social legal or financial), failure to fulfill roles, concern expressed about their drug use and if the person has ever tried and failed to control their drug use. Finally, injection of drugs was assessed. The standard ASSIST scoring algorithm was used to calculate a score for ATS use (range 0-39) [20].

We assessed secondary outcomes in terms of: (i) help-seeking intentions (general help-seeking questionnaire (GHSQ)) [21]; actual help-seeking questionnaire (AHSQ) [22, 23]; (ii) readiness to change, modified to assess ATS rather than alcohol (Readiness to Change Questionnaire (RTCQ) [24]); (iii) psychological distress (Kessler 10 [25]); (iv) poly-drug use measured by the ASSIST [20]; (v) days out of role [26]; and (vi) and quality of life (European Health Interview Survey (EUROHIS) Quality of Life scale [27]). We also collected demographic information (e.g., age, sex, marital status), drug use history (e.g. age of first use of ATS) and severity of dependence (Severity of Dependence Scale [28]).

The RTCQ has four items relating to each of the stages: 'pre-contemplation'; 'contemplation'; and 'action'. The five point scales were summed to obtain scores for each stage, with participants designated to their highest scoring stage, or in the event to tied scores, the higher stage [24]. Psychological distress was indexed as the total score (range 10-50) on the K-10 [25]. Poly-drug use was the sum of ASSIST classes of drugs endorsed, excluding ATS use [20].The GHSQ asked "How likely is it that you would seek help from each of the following people for any amphetamine or other drug use problems during the next 4 weeks?" and provided a list of nine potential sources of help (e.g. friend, mental health professional, other). The seven point scale ranged from extremely unlikely (1) to extremely likely (7). The AHSQ asked "Which of the following people have you gone to for advice or help in the past 2 weeks for any amphetamine or other drug use problems?" and listed the same nine sources as the GHSQ. 'Days out of role' was based on Kessler's questions but referencing "ATS drug use (e.g. methamphetamine, ecstasy, ice)" rather than "depression" [26] and quality of life was the total EUROHIS score [27].
"

6a-i) Online questionnaires: describe if they were validated for online use and apply CHERRIES items to describe how the questionnaires were designed/deployed

Not validated for online use

6a-ii) Describe whether and how "use" (including intensity of use/dosage) was defined/measured/monitored

"each page in a module had to be opened in sequence to complete the module and obtain access to the next one." Use was defined as opening each element of a module

6a-iii) Describe whether, how, and when qualitative feedback from participants was obtained

Qualitative feedback was obtained in the development stage as described in the protocol paper.

6b) CONSORT: Any changes to trial outcomes after the trial commenced, with reasons

No changes

7a) CONSORT: How sample size was determined

7a-i) Describe whether and how expected attrition was taken into account when calculating the sample size

"The study was designed to detect a medium effect (e.g. d = 0.5) [29] with power of 0.8, which requires a sample of 60 people per group: to allow for 20% attrition we recruited 80 people per group. In estimating the sample size, we drew on findings for stimulant users who were recruited in primary care settings and received a brief intervention in the ASSIST development study [20]. That group may be less heterogeneous than the current sample"

7b) CONSORT: When applicable, explanation of any interim analyses and stopping guidelines

There were interim analyses or stoping criteria

8a) CONSORT: Method used to generate the random allocation sequence

"We used a simple randomization process that was fully automated"

8b) CONSORT: Type of randomisation; details of any restriction (such as blocking and block size)

"We used a simple randomization process that was fully automated with permuted blocks of four"

9) CONSORT: Mechanism used to implement the random allocation sequence (such as sequentially numbered containers), describing any steps taken to conceal the sequence until interventions were assigned

Fully automated

10) CONSORT: Who generated the random allocation sequence, who enrolled participants, and who assigned participants to interventions

Fully automated

11a) CONSORT: Blinding - If done, who was blinded after assignment to interventions (for example, participants, care providers, those assessing outcomes) and how

11a-i) Specify who was blinded, and who wasn't

"The analysis was conducted blind to study condition up to the 'per protocol' analysis by RJT."

11a-ii) Discuss e.g., whether participants knew which intervention was the "intervention of interest" and which one was the "comparator"

The study used a wait list control group so all participants would have been aware of which group they were in.

11b) CONSORT: If relevant, description of the similarity of interventions

N/A

12a) CONSORT: Statistical methods used to compare groups for primary and secondary outcomes

We used generalised estimating equations with repeated measure: the critical assessment was the group by time interaction for the primary and secondary outcome measures.

12a-i) Imputation techniques to deal with attrition / missing values

The initial analysis was intention to treat with no imputation.
:A sensitivity analysis was conducted using multiple imputation of missing data using fully conditional specification with an iterative Markov chain Monte Carlo (MCMC) method"
"Finally a 'per protocol' analysis was conducted to evaluate the effect of completing at least one module of the intervention"

12b) CONSORT: Methods for additional analyses, such as subgroup analyses and adjusted analyses

"Finally a 'per protocol' analysis was conducted to evaluate the effect of completing at least one module of the intervention"

All analyses included baseline severity of dependence and time varying 'actual help-seeking' as adjustments

RESULTS

13a) CONSORT: For each group, the numbers of participants who were randomly assigned, received intended treatment, and were analysed for the primary outcome

Yes, see figure 1 consort flow diagram

13b) CONSORT: For each group, losses and exclusions after randomisation, together with reasons

Yes, see figure 1 consort flow diagram
However, no participants 'withdrew' from the study so there was no documentation of reasons for 'loss to follow-up' (e.g. participants just didn't complete follow-up.

13b-i) Attrition diagram

No diagram other than the consort figure. From text "From the 81 intervention participants, 51 (63%) completed, 13 (16%) started and 17 (21%) did not attempt the first module. The second module was completed by 45 (56%) participants and started by another two (2.5%): the respective figures for the third module were 39 (48%) and four (5%)."

"The proportion who submitted follow-up data in the intervention group varied with the number of modules completed (seven (23%) who completed no modules, two (33%) who completed one module, four (67%) who completed two modules and 22 (56%) who completed all three: ...."

14a) CONSORT: Dates defining the periods of recruitment and follow-up

Recruitment opened in January 2013 and closed in July 2013.

14a-i) Indicate if critical "secular events" fell into the study period

We are not aware of any events that would have particularly influenced ATS use (or desire to cease use)

14b) CONSORT: Why the trial ended or was stopped (early)

N/A

15) CONSORT: A table showing baseline demographic and clinical characteristics for each group

Yes, see table 1

15-i) Report demographics associated with digital divide issues

Table 1 includes age, gender education and employment

16a) CONSORT: For each group, number of participants (denominator) included in each analysis and whether the analysis was by original assigned groups

16-i) Report multiple "denominators" and provide definitions

This is done at a general level - e.g. n who completed follow-up: multiple imputation and per protocol analysis. However, those missing data on a specific variable are not reported.

16-ii) Primary analysis should be intent-to-treat

The primary analysis was ITT

17a) CONSORT: For each primary and secondary outcome, results for each group, and the estimated effect size and its precision (such as 95% confidence interval)

Yes see table 2

17a-i) Presentation of process outcomes such as metrics of use and intensity of use

"From the 81 intervention participants, 51 (63%) completed, 13 (16%) started and 17 (21%) did not attempt the first module. The second module was completed by 45 (56%) participants and started by another two (2.5%): the respective figures for the third module were 39 (48%) and four (5%). Thus, 39 (48%) completed all the modules, six (7%) completed two modules and six completed one module."

17b) CONSORT: For binary outcomes, presentation of both absolute and relative effect sizes is recommended

N/A

18) CONSORT: Results of any other analyses performed, including subgroup analyses and adjusted analyses, distinguishing pre-specified from exploratory

All the major (e.g. ITT) analyses were pre-specified. Descriptive analysis were not specified nor were exploratory analysis of retention and engagement.

18-i) Subgroup analysis of comparing only users

"Note: analysis of 'per protocol' data does not represent randomized outcomes"

19) CONSORT: All important harms or unintended effects in each group

No harms or unintended effects identified

19-i) Include privacy breaches, technical problems

No harms or unintended effects identified

19-ii) Include qualitative feedback from participants or observations from staff/researchers

None available

DISCUSSION

20) CONSORT: Trial limitations, addressing sources of potential bias, imprecision, multiplicity of analyses

20-i) Typical limitations in ehealth trials

"There are a number of limitations that need to be acknowledged in the interpretation of these findings. The sample would be regarded as having less severe substance use problems, ...Thus, care should be taken in extrapolating beyond this type of ATS user. Nevertheless, 57 participants scored five or more on the SDS and on the basis of this screening measure, are likely to be ATS dependent [36]. The loss to follow-up of a significant proportion of participants threatens the internal validity of the study. Although this was not related to group allocation in a logistic model, the association with increased severity of dependence and poly-drug use, reinforces the caveat that this type of low intensity intervention may not be suitable for those with more severe drug use problems....,""

"Other e-health interventions with illicit drug users (cocaine) have encountered more extensive attrition [15] but the results obtained in the current study are comparable with in-person interventions for ATS [6] and consistent with the broader literature from fully automated internet interventions [38]. We also identified significant differences in follow-up between the study groups which may also bias the results."

"A further concern is the low level of engagement with the intervention: .... future research is required to investigate ways to encourage intervention completion. This is particularly important given previous findings that completion of at least one in-person module of a four-session intervention for ATS was associated with superior ATS reductions than those who did not return for any sessions..... Finally, the low level of engagement diminishes any potential difference between the study groups."

21) CONSORT: Generalisability (external validity, applicability) of the trial findings

21-i) Generalizability to other populations

"The sample would be regarded as having less severe substance use problems, ...Thus, care should be taken in extrapolating beyond this type of ATS user. ...The loss to follow-up of a significant proportion of participants threatens the internal validity of the study. ...., reinforces the caveat that this type of low intensity intervention may not be suitable for those with more severe drug use problems....,""

"Other e-health ..... more extensive attrition.... We also identified significant differences in follow-up between the study groups which may also bias the results."

"A further concern is the low level of engagement with the intervention: .... future research is required to investigate ways to encourage intervention completion. ....., the low level of engagement diminishes any potential difference between the study groups.""

21-ii) Discuss if there were elements in the RCT that would be different in a routine application setting

Not addressed due to the low impact of the study (e.g. not now intended for general release at the this stage). However, we do suggest further work on approach to improve its effectiveness.

22) CONSORT: Interpretation consistent with results, balancing benefits and harms, and considering other relevant evidence

22-i) Restate study questions and summarize the answers suggested by the data, starting with primary outcomes and process outcomes (use)

"To the best of our knowledge, this is the first Internet intervention developed specifically for users of ATS."

"There was only one (time by group) significant change on any of the key outcome measures...., . Further, the effect sizes for the intervention were smaller than those estimated in the design phase. The findings of the multiple imputations analysis reinforce the conclusion that the study was 'insufficiently powered' to detect small effects."
" the high level of attrition and low level of engagement limit the conclusions that can be draw from these data. "

22-ii) Highlight unanswered new questions, suggest future research

"Improving engagement is a critical goal for interventions with substance using groups."

Other information

23) CONSORT: Registration number and name of trial registry

Yes
"Trial Registration: Australian and New Zealand Clinical Trials Registry (www.anzctr.org.au/) ACTRN12611000947909"

24) CONSORT: Where the full trial protocol can be accessed, if available

Not available

25) CONSORT: Sources of funding and other support (such as supply of drugs), role of funders

"This study was funded by The Commonwealth of Australia, Department of Health and Ageing. Helen Christensen is funded by NHMRC Fellowship 1056964. Kathleen Griffiths is funded by NHMRC Fellowship 525413. Frances Kay-Lambkin is funded by NHMRC Fellowship 1008972. Robert Tait is funded by a Curtin University Research Fellowship"

X26-i) Comment on ethics committee approval

"The study received approval from The Australian National University Human Research Ethics committee and was registered with the Australian and New Zealand Clinical Trials Registry (www.anzctr.org.au/) ACTRN 12611000947909."

x26-ii) Outline informed consent procedures

"Eligible participants provided active consent by 'clicking' on a box for each element of the consent form (appendix 1). "

X26-iii) Safety and security procedures

" We also provided all participants with contact details for emergency services, such as Lifeline Australia"

"Participants who were not eligible for the study were provided with information about other potentially useful websites and resources."

X27-i) State the relation of the study team towards the system being evaluated

None of the authors have any financial conflict of interests to declare with respect to this study. However, the team who evaluated the intervention were also involved in its development.
